# Supplementary material for: Cytotoxic Mechanism of Momilactones A and B against Acute Promyelocytic Leukemia and Multiple Myeloma Cell Lines
Source: Cancers (Basel). 2022 Oct 4;14(19):4848. doi: 10.3390/cancers14194848 (PMC9564300; doi:10.3390/cancers14194848)
Supplement: Supplementary file 1 [file cancers-14-04848-s001.zip › cancers-1779091-supplementary.pdf]

cancers

MDPI

Article

Cytotoxic Mechanism of Momilactones A and B against Acute Promyelocytic Leukemia and Multiple Myeloma Cell Lines

Supplementary materials:

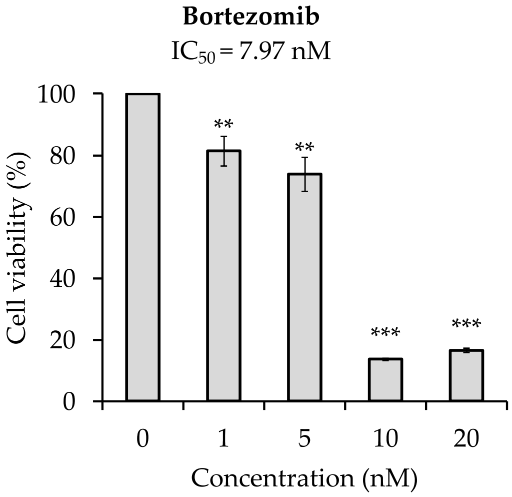

Figure S1. Effects of bortezomib on cell viability of multiple myeloma (U266) cell lines after 48 h. $^{**}p < 0.01$ versus control (0 nM); $^{***}p < 0.001$ versus control (0 nM).

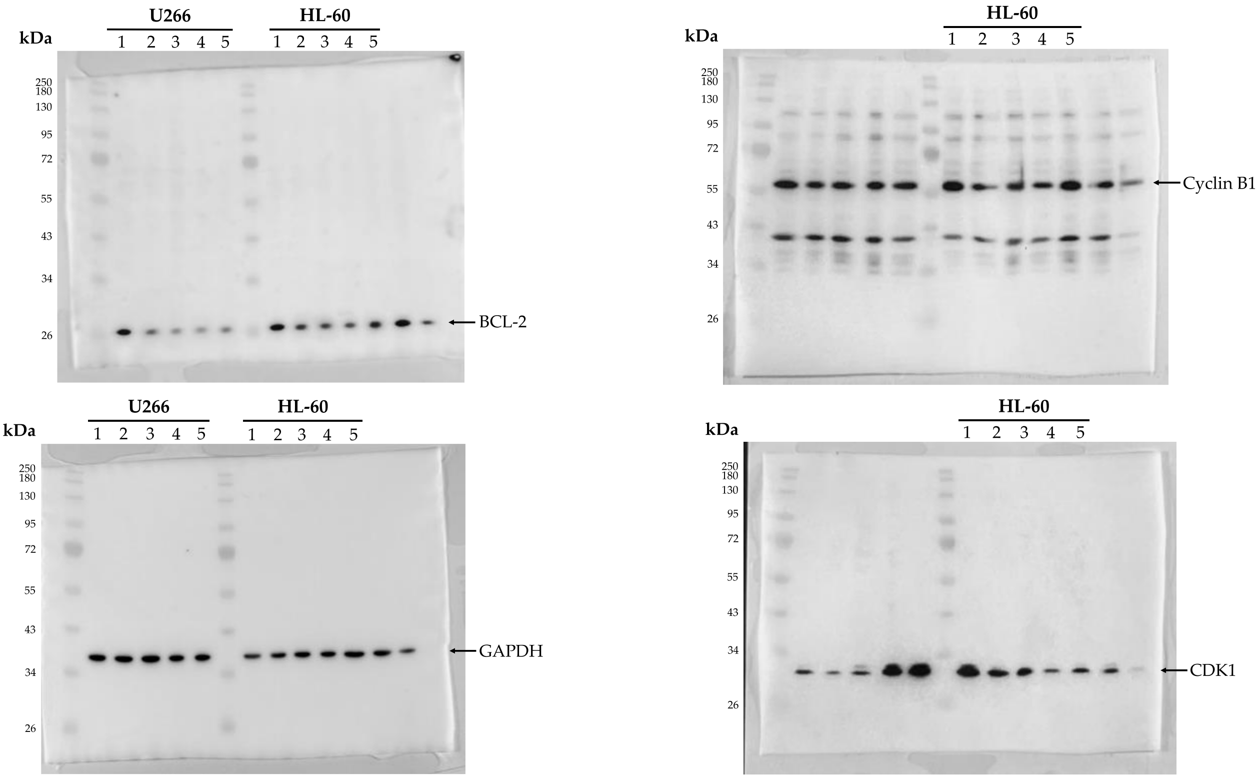

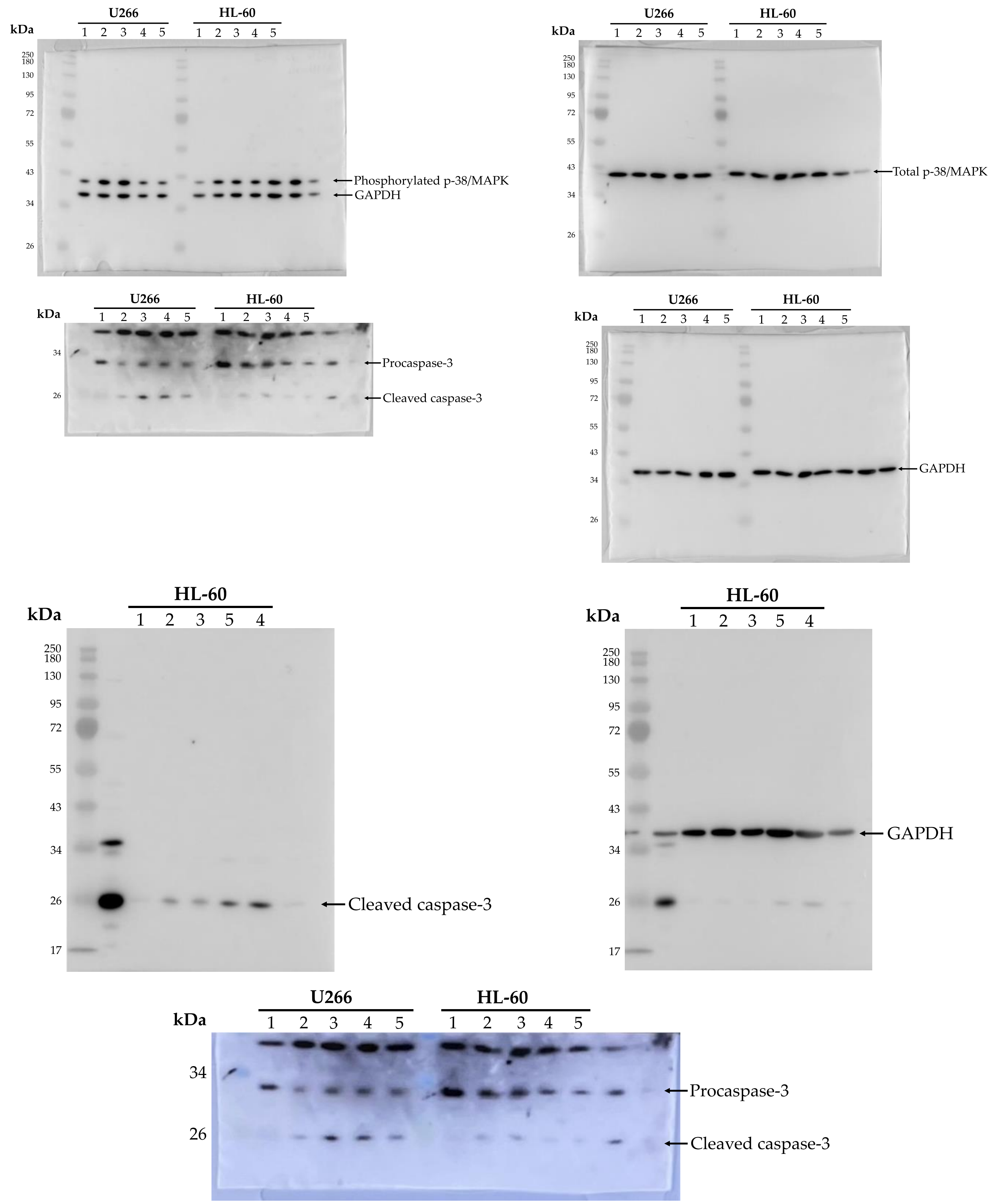

Figure S2. Western blotting results. 1, Ctrl; 2, MB-24 h; 3, MB-48 h; 4, MAB-48 h; 5, MAB-24 h.
